# Identification of Type 2 Diabetes Based on a Ten-Gene Biomarker Prediction Model Constructed Using a Support Vector Machine Algorithm

**DOI:** 10.1155/2022/1230761

**Published:** 2022-03-04

**Authors:** Jiabin Li, Jieying Ding, D. U. Zhi, Kaiyun Gu, Hui Wang

**Affiliations:** ^1^Department of Pharmacy, Zhejiang University School of Medicine Children's Hospital, Hangzhou, China 310052; ^2^Department of National Center, Zhejiang University School of Medicine Children's Hospital, Hangzhou, China 310052; ^3^Laboratory and Equipment Management Office, Zhejiang University of Traditional Chinese Medicine, Hangzhou, China 310052

## Abstract

**Background:**

Type 2 diabetes is a major health concern worldwide. The present study is aimed at discovering effective biomarkers for an efficient diagnosis of type 2 diabetes.

**Methods:**

Differentially expressed genes (DEGs) between type 2 diabetes patients and normal controls were identified by analyses of integrated microarray data obtained from the Gene Expression Omnibus database using the Limma package. Functional analysis of genes was performed using the R software package clusterProfiler. Analyses of protein-protein interaction (PPI) performed using Cytoscape with the CytoHubba plugin were used to determine the most sensitive diagnostic gene biomarkers for type 2 diabetes in our study. The support vector machine (SVM) classification model was used to validate the gene biomarkers used for the diagnosis of type 2 diabetes.

**Results:**

GSE164416 dataset analysis revealed 499 genes that were differentially expressed between type 2 diabetes patients and normal controls, and these DEGs were found to be enriched in the regulation of the immune effector pathway, type 1 diabetes mellitus, and fatty acid degradation. PPI analysis data showed that five MCODE clusters could be considered as clinically significant modules and that 10 genes (*IL1B*, *ITGB2*, *ITGAX*, *COL1A1*, *CSF1*, *CXCL12*, *SPP1*, *FN1*, *C3*, and *MMP2*) were identified as “real” hub genes in the PPI network using algorithms such as Degree, MNC, and Closeness. The sensitivity and specificity of the SVM model for identifying patients with type 2 diabetes were 100%, with an area under the curve of 1 in the training as well as the validation dataset.

**Conclusion:**

Our results indicate that the SVM-based model developed by us can facilitate accurate diagnosis of type 2 diabetes.

## 1. Introduction

Diabetes mellitus (DM) is one of the top three major chronic noncommunicable diseases [1]. Over 90% of DM cases are those of type 2 diabetes. In recent years, the incidence of type 2 diabetes has been increasing significantly each year [2]. According to the latest survey released by the International Diabetes Federation in 2019, diabetes has a prevalence of 9.3%, and it affects approximately 463 million adults worldwide. It is expected that the number of affected individuals will reach 578 million (10.2%) by 2030 and 700 million (10.9%) by 2045 [3], making the prevention and treatment of type 2 diabetes a serious challenge that faces humanity.

The support vector machine (SVM) algorithm was first proposed by Vapnik et al. [4] in 1995 as a supervised learning method in machine learning, and it gradually developed and matured to have a wide range of applications in the mid-1990s. Later on, a series of improved and extended algorithms stemming from SVM were developed, such as multiclass SVM classification, support vector regression, least-squares SVM, support vector clustering, and semisupervised SVM [5].

Machine learning tools are used to detect key features from complex datasets. SVM is a machine learning tool that is widely used in disease research to build predictive models, and it is known to produce effective and predictable models [6–9]. The following are a few examples of efficient predictive models built using SVM: a study showing the application of an SVM-based approach to identify postmenopausal women with low bone density [10] and a gene signature associated with postmenopausal osteoporosis that was detected and validated using SVM [11].

The Gene Expression Omnibus (GEO) [12], an online public database made available by National Center for Biotechnology Information in 2000, is currently one of the most comprehensive gene expression databases. Based on data from this database, we systematically analyzed the expression patterns of genes associated with type 2 diabetes samples at a transcriptional level.

Based on the literature study [13, 14], we hypothesize that multigene panels may be more effective and comprehensive in predicting the prognosis of type 2 diabetes patients. Therefore, we attempted to identify and verify a robust prognostic feature that predicts survival rate by integrating multiple datasets of type 2 diabetes patients. In this study, we constructed a risk prediction model based on SVM at the transcription level for type 2 diabetes patients; this model may supplement traditional clinical prognostic factors and further provide more effective therapeutic intervention and personalized treatment for type 2 diabetes patients.

## 2. Materials and Methods

### 2.1. Data Acquisition

MINiML formatted family files of type 2 diabetes-related microarray datasets, GSE164416 [15], GSE156993 [16], GSE161355 [17], GSE163980, GSE76895 [18], GSE9006 [19], and GSE78721 [20], were downloaded from GEO. Those datasets we use are the processed data from the GEO database, which has been background processed and normalized.

Type 2 diabetes samples and control samples were retrieved from the GEO dataset, and the probe IDs were converted to gene symbols. Any single probe corresponding to multiple genes was removed, and for genes corresponding to multiple gene symbols, the median gene expression was considered.

The RNA-Seq dataset (GSE164416) was processed using the following steps.

Type 2 diabetes samples and control samples from the dataset were retrieved, and the expression spectrum was counted in terms of transcripts per million. ENSG IDs were converted into gene symbols, and the median value was considered for expression of genes corresponding to multiple gene symbols.

Clinical statistics of the processed samples are shown in Table 1. Clinical information of datasets is shown in Table 2.

### 2.2. Identification of Differentially Expressed Genes (DEGs)

The “Limma” package [21] in the latest R language (version 4.2.2) was used to screen DEGs between the type 2 diabetes samples and the normal control samples, and the criteria for statistical significance were ∣log_2_FC | >1 and FDR < 0.05.

### 2.3. Functional Enrichment Analysis

Kyoto Encyclopedia of Genes and Genomes (KEGG) pathway analysis and Gene Ontology (GO) functional enrichment analysis based on cellular components (CCs), biological processes (BPs), and molecular functions (MFs) were performed on the DEGs using the R software package clusterProfiler [22]. *P* < 0.05 was used as the significance threshold for enrichment.

### 2.4. Protein-Protein Interaction (PPI) Networks

PPI networks facilitate the study of the molecular mechanisms of a disease from a system perspective, for example, in the discovery of new drug targets. We constructed a PPI network using resources from the STRING database [23], a database of known and predicted protein-protein interactions. The PPI network of the modular gene was obtained by importing the module genes into the STRING database and processing them with the resources available on the database website. Generally, the importance of a network node is positively correlated with the node having more connections to the genes in the network, thus implying the node to have a greater connectivity across the network. Each point in the PPI network for screening the modular pivot genes was calculated using the Cytoscape (version 3.7.2) [24] software with the plugin CytoHubba [25] and using the following four algorithms: Degree, MCC (Maximal Clique Centrality), Closeness, and Betweenness.

### 2.5. Construction of a Support Vector Machine (SVM) Classifier

SVM is a supervised machine learning classification algorithm that distinguishes sample types by estimating the chance of a sample belonging to a certain class. For the training set, the SVM classifier was constructed using the SVM method based on the optimal mRNA set in the R package e1071 (version 1.6-8, http://cran.r-project.org/web/packages/e1071).

The performance of the SVM classifier was evaluated in the training and validation sets by the area under the curve (AUC) of the receiver operating characteristic (ROC) curve as an evaluation metric.

## 3. Results

### 3.1. Identification and Functional Annotation of DEGs

**Figure 1:**
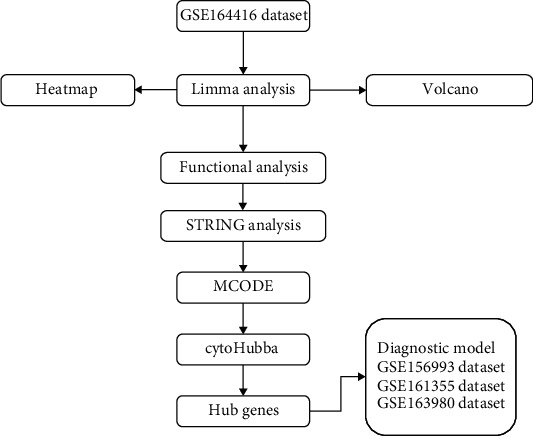
Workflow chart.

**Figure 2:**
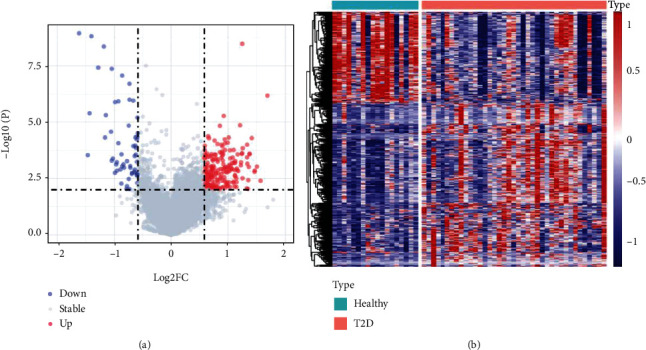
Identification of differentially expressed genes. (a) The volcano map of differentially expressed genes in the GSE164416 dataset. (b) The heat map of differentially expressed genes in the GSE164416 dataset. *P* < 0.01 and ∣log_2_FC | >1.5.

**Figure 3:**
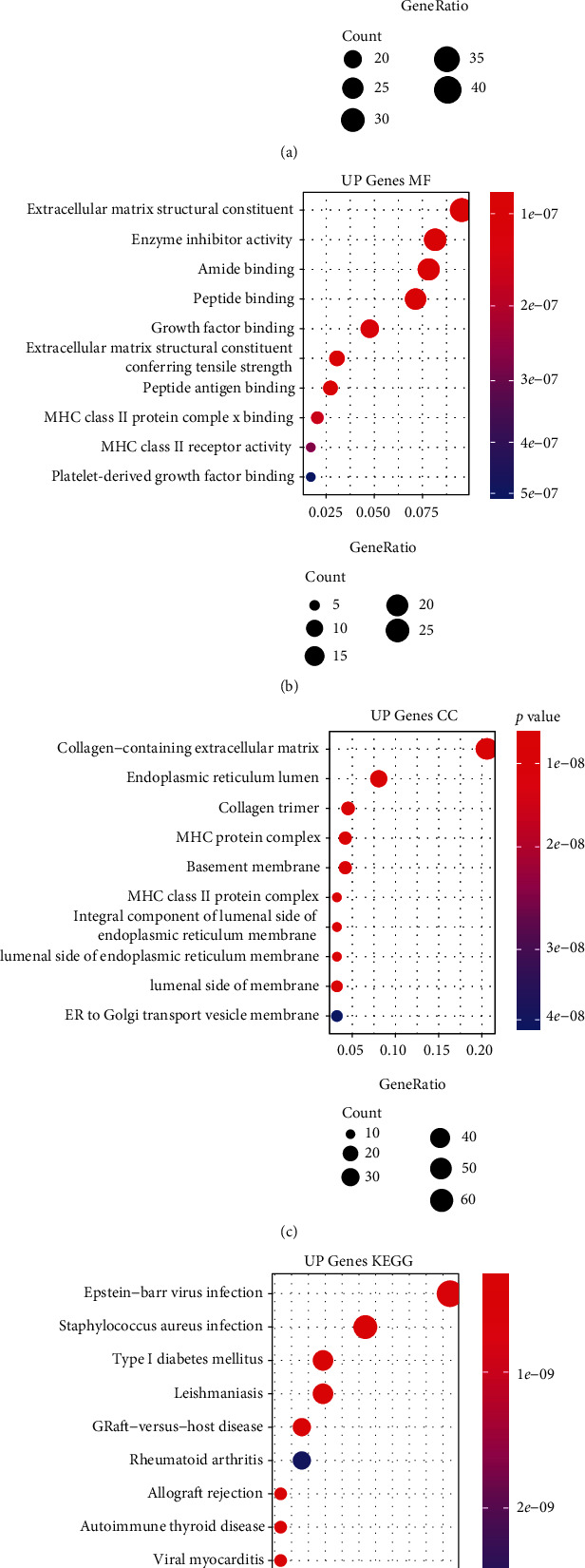
Functional enrichment analysis of differentially expressed upregulated genes. (a) The biological process annotation map of differentially expressed upregulated genes. (b) The cellular component annotation map of differentially expressed upregulated genes. (c) The molecular function annotation map of differentially expressed upregulated genes. (d) The Kyoto Encyclopedia of Genes and Genomes annotation map of differentially expressed upregulated genes.

**Figure 4:**
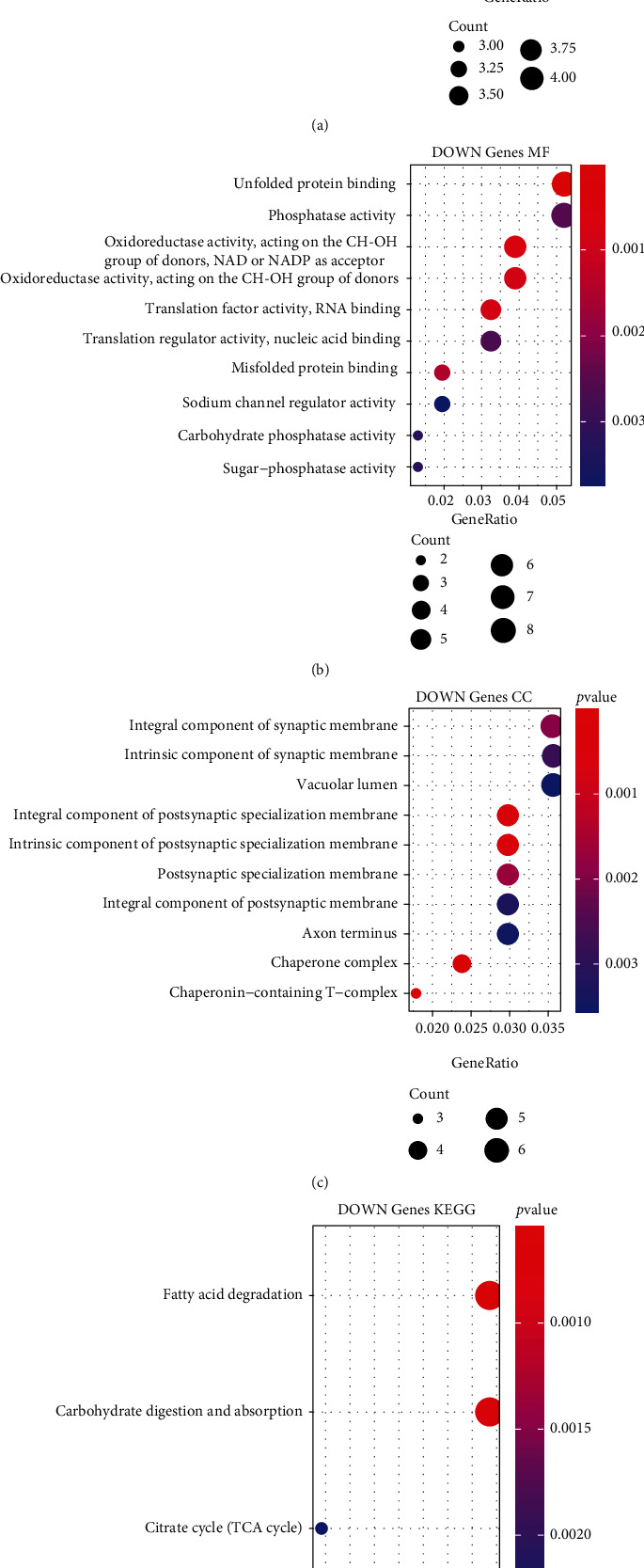
Functional enrichment analysis of differentially expressed downregulated genes. (a) The biological process annotation map of differentially expressed downregulated genes. (b) The cellular component annotation map of differentially expressed downregulated genes. (c) The molecular function annotation map of differentially expressed downregulated genes. (d) The Kyoto Encyclopedia of Genes and Genomes annotation map of differentially expressed downregulated genes.

The analysis flowchart describing the methodology of this study is shown in Figure 1. A total of 499 DEGs were obtained, of which, 320 were upregulated and 179 were downregulated in the type 2 diabetes samples (Figures 2(a) and 2(b)). Furthermore, KEGG pathway analysis and GO functional enrichment analysis were performed on the 499 DEGs using the R software package clusterProfiler. For the 320 upregulated genes, the top 10 significantly enriched BPs, MFs, and CCs are shown in Figures 3(a)–3(c). Based on the KEGG annotation, 63 pathways were obtained, which included pathways associated with Epstein-Barr virus infection, type I diabetes mellitus, and *Staphylococcus aureus* infection (Figure 3(d)). For the 179 downregulated genes, the top 10 significantly enriched BPs, MFs, and CCs are shown in Figures 4(a)–4(c). Based on the KEGG annotation, three pathways were obtained, which included fatty acid degradation, carbohydrate digestion and absorption, and the citric acid cycle (Figure 4(d)).

### 3.2. Protein-Protein Interaction Networks

**Figure 5:**
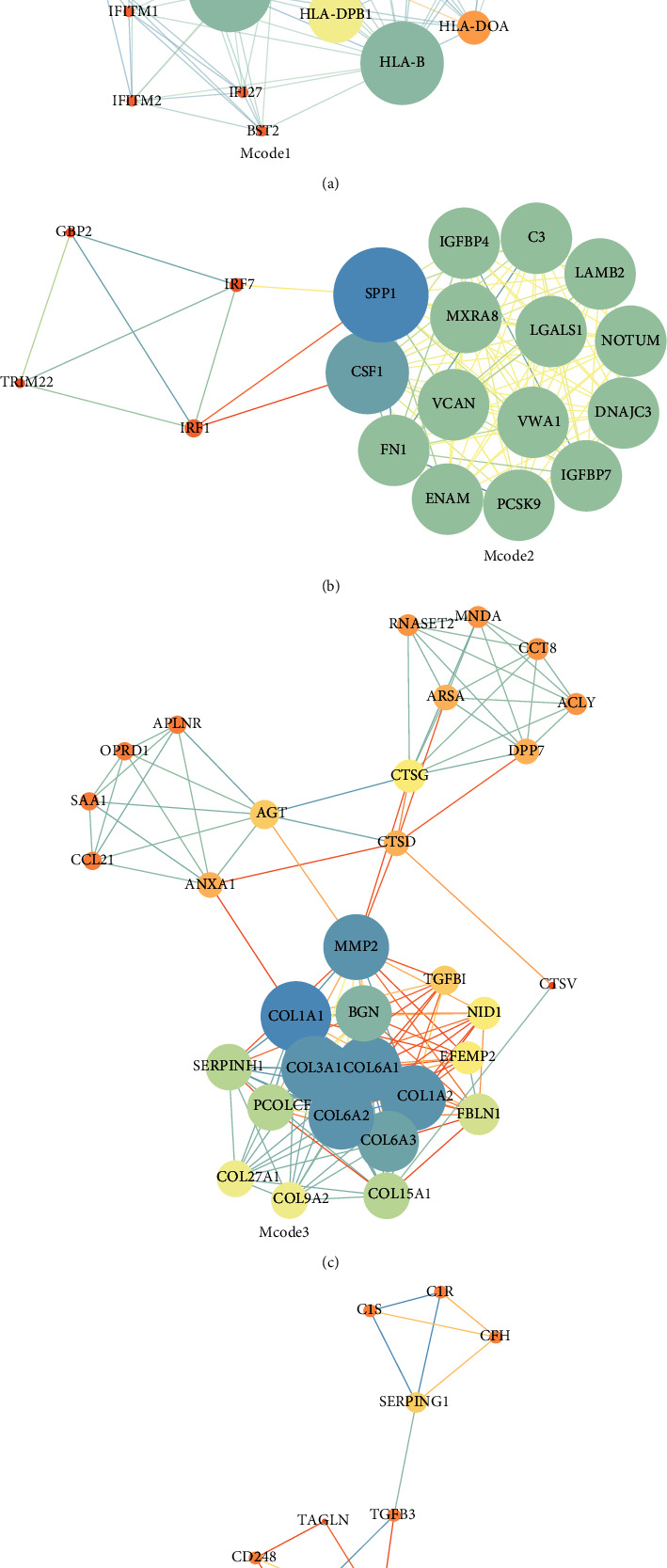
Gene protein-protein interaction maps of the functional modules mined by MCODE.

The PPI network of the 499 DEGs was constructed using the STRING database. Cytoscape (version 3.7.2) was used to filter the network modules, and the MCODE algorithm plugin found five clinically significant modules (Figure 5).

### 3.3. Identification of Hub Genes

**Figure 6:**
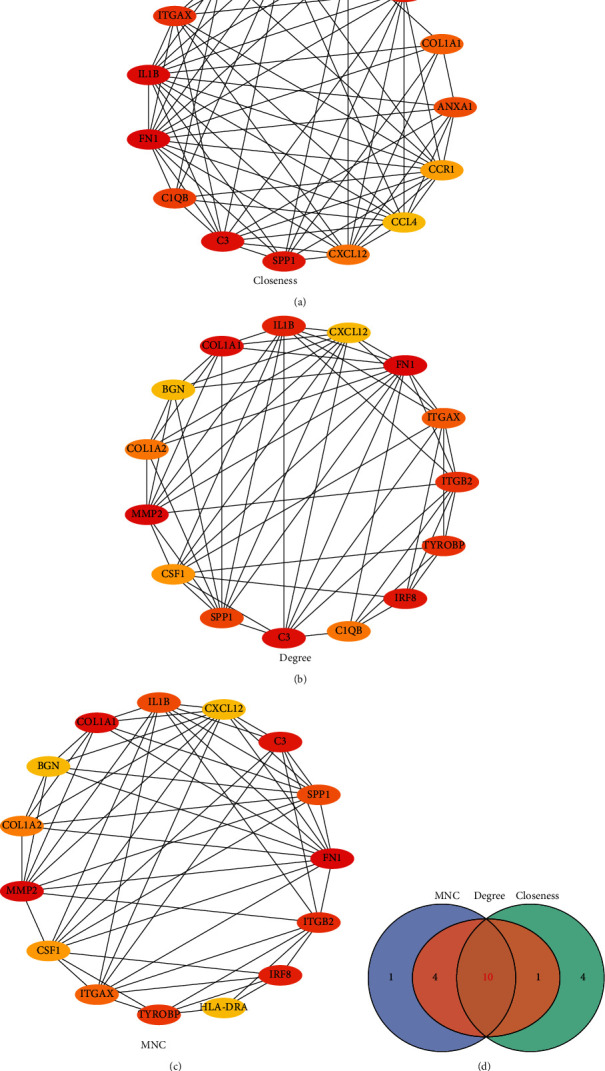
Identification of hub genes. (a) Protein-protein interaction network diagram of hub genes identified by the Closeness algorithm. (b) Protein-protein interaction network diagram of hub genes identified by the Degree algorithm. (c) Protein-protein interaction network diagram of hub genes identified by the MNC algorithm. (d) Venn diagram of genes obtained by the three algorithms.

Calculations were performed on the PPI network developed with the 499 DEGs using the three algorithms, Degree, MNC, and Closeness, available in CytoHubba, a plugin of the Cytoscape (version 3.7.2) software, to select the top 15 genes as the key genes (Figures 6(a)–6(c)). The intersection of the hub genes obtained by the three algorithms was considered to contain the ten “real” hub genes (*IL1B*, *ITGB2*, *ITGAX*, *COL1A1*, *CSF1*, *CXCL12*, *SPP1*, *FN1*, *C3*, and *MMP2*) (Figure 6(d)).

### 3.4. Construction and Validation of the Diagnostic Model

**Figure 7:**
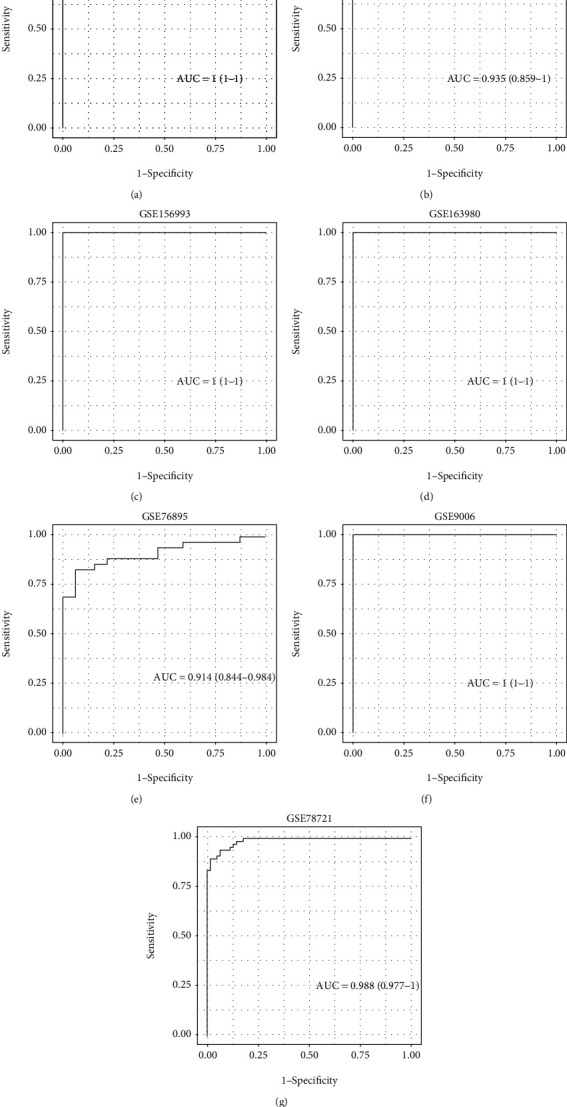
Construction and validation of diagnostic models. (a) Receiver operating characteristic curves of the classification results of the diagnostic model on the samples of the GSE164416 dataset. (b) Receiver operating characteristic curves of the classification results of the diagnostic model on the samples of the GSE161355 dataset. (c) Receiver operating characteristic curves of the classification results of the diagnostic model on the samples of the GSE156993 dataset. (d) Receiver operating characteristic curves of the classification results of the diagnostic model on the samples of the GSE163980 dataset. (e) Receiver operating characteristic curves of the classification results of the diagnostic model on the samples of the GSE76895 dataset. (f) Receiver operating characteristic curves of the classification results of the diagnostic model on the samples of the GSE9006 dataset. (g) Receiver operating characteristic curves of the classification results of the diagnostic model on the samples of the GSE78721 dataset.

The GSE164416 dataset was used as the training dataset, and the datasets GSE156993, GSE161355, and GSE163980 were used as validation datasets. The ten “real” hub genes served as features in the training dataset, and their corresponding expression profiles were obtained, according to which the SVM classification model was constructed (1000 iterations and 10-fold cross-validation). The classification accuracy of the GSE156993 dataset was 100%, as 57 out of 57 samples were correctly classified; the sensitivity and specificity of the model were both 100%, with an AUC of the ROC curve of 1 (Figure 7(a)). Furthermore, all samples in the GSE156993, GSE161355, and GSE163980 datasets were correctly classified, showing the model to have a high classification accuracy, with both the sensitivity and specificity being 100% and the AUC of the ROC curve being 1 (Figures 7(b)–7(e)). The three datasets (GSE76895, GSE9006, and GSE78721) were further verified by our diagnostic model. The results showed that in the GSE76895 dataset, 62 out of 68 samples were correctly classified, with a classification accuracy of 91.2%. The sensitivity is 88.9% and specificity is 93.8%, and the area under the ROC curve was 0.914 (Figure 7(e)). In the GSE9006 dataset, 36 of the 36 samples were correctly classified with a classification accuracy of 100%. The sensitivity and specificity of the model were 100%, and the area under the ROC curve was 1 (Figure 7(f)). In the GSE78721 dataset, 128 of the 130 samples were correctly classified, with a classification accuracy of 98.5%. The sensitivity and specificity of the model were 100% and 96.8%, and the area under the ROC curve was 0.988 (Figure 7(g)).

## 4. Discussion

The present study identified 499 DEGs between type 2 diabetes patients and normal controls from the GSE164416 dataset. PPI analysis identified five modules as being clinically significant, and subsequent analysis revealed 10 genes (*IL1B*, *ITGB2*, *ITGAX*, *COL1A1*, *CSF1*, *CXCL12*, *SPP1*, *FN1*, *C3*, and *MMP2*) to be the “real” hub genes. The SVM-based classification involving the 10-gene signature achieved a 100% prediction accuracy in distinguishing patients with type 2 diabetes from normal controls with 100% sensitivity, 100% specificity, and an AUC of 1. The validation results obtained using the other three datasets (GSE156993, GSE161355, and GSE163980) further support the validity of our model.

Tyler et al. developed an inference method based on a general model of molecular, neuronal, and ecological oscillatory systems that merges the advantages of both model-based and model-free methods, namely, accuracy, broad applicability, and usability [26]. SVM methods have been widely used in classification and prediction owing to their feasibility of extracting higher order statistics. Abbas et al. discovered a type 2 diabetes prediction model based on the features derived only from the plasma glucose concentrations measured during an oral glucose tolerance test using SVM [27]. Cui et al. indicated that the proposed SVM-based method achieved an accuracy of 81.02%, a sensitivity of 82.89%, and a specificity of 79.23%, and it outperformed other popular algorithms in identifying diabetic patients who may be readmitted [28]. An SVM algorithm was used to classify osteoporosis in patients with type 2 diabetes by relying on several serological items and personal information with a diagnostic accuracy of 88% [29]. The present study shows the application of SVM based on a 10-gene signature to identify patients with type 2 diabetes by distinguishing positive type 2 diabetes samples from normal control samples with a high sensitivity.

Type 2 diabetes is a multifactorial, typical complex disease, which is associated with lifestyle and other environmental factors [30, 31]. In this research, a set of ten “real” hub genes, *IL1B*, *ITGB2*, *ITGAX*, *COL1A1*, *CSF1*, *CXCL12*, *SPP1*, *FN1*, *C3*, and *MMP2*, was integrated into a model to predict type 2 diabetes. Studies have shown these genes to be involved in the development of type 2 diabetes, for example, a study showing IL-1*β*-induced *β* cell dysfunction [32]. Glawe et al. showed that genetic deficiency of *ITGB2* completely prevented the development of hyperglycemia and frank diabetes in NOD mice [33]. Some experiments have found that islet inflammation could promote beta cell dysfunction in type 2 diabetes with increased expression of *ITGAX* [34, 35]. *COL1A1* was the most significant gene in the extracellular matrix-receptor interaction pathway and was linked to hypoglycemic activity for the first time. Thus, *COL1A1* is a novel potential therapeutic target for alleviating type 2 diabetes. It is reported that CSF1 receptor dephosphorylation inhibits alveolar bone resorption in diabetic periodontitis [36]. Genetic variations of the *CXCL12* gene might affect trafficking of inflammatory cells or defected precursors and hence induce tendencies toward diabetic complications. The SDF1-3′A genetic variation of *CXCL12* influences the development of late vascular diabetic complications, and study reported that this genetic variation regulates the expression of *CXCL12* [37]. *SPP1* encodes osteopontin which has been shown to cause glomerular damage and interstitial fibrosis in diabetic kidney disease [38]. A study involving signaling pathway enrichment analysis reported that ECM-receptor interaction is one of the main pathways in the diabetic nephropathy extracellular matrix and that *FN1* is involved in the ECM-receptor interaction pathway [39]. Human pancreatic islets highly express C3 and are associated with the donor status of type 2 diabetes. C3 may be upregulated as a cytoprotective factor during type 2 diabetes to combat *β* cell dysfunction caused by impaired autophagy [40]. Type 2 diabetes increases the activity of matrix metalloproteinases (MMPs) [41]. Clinical correlation studies suggest that high circulating MMP-2 levels may correlate with the severity of periodontitis in type 2 diabetes [42]. Further research is required to explore the roles of the 10 “real” hub genes identified in our study in type 2 diabetes.

In summary, the present study used PPI analysis to identify 10 hub genes associated with type 2 diabetes. In addition, the components of the combination of these 10 genes may serve as potential biomarkers for type 2 diabetes. However, the lack of a detailed biological investigation and the lack of validation with a larger sample size were considered as limitations of this study. Further studies are therefore needed before clinical application to verify the diagnostic ability of this 10-gene signature for type 2 diabetes.

## 5. Conclusion

In this study, we analyzed transcriptional level gene expression data using SVM to construct a risk prediction model for type 2 diabetes patients, which may supplement traditional clinical prognostic factors, enabling clinicians to provide more effective therapeutic intervention and personalized treatment for type 2 diabetes patients.

## Figures and Tables

**Table 1:** Clinical sample information of datasets.

Datasets	Expression	Platforms
GSE164416		
Healthy	18	GPL16791
T2D	39	
GSE156993		
Healthy	6	GPL570
T2D	12	
GSE161355		
Healthy	15	GPL570
T2D	18	
GSE163980		
Healthy	5	GPL20115
T2D	5	
GSE76895		
Healthy	32	GPL570
T2D	36	
GSE9006		
Healthy	24	GPL96
T2D	12	
GSE78721		
Healthy	62	GPL15207
T2D	68	

**Table 2:** Clinical feature information of GEO datasets.

	*b*	GSE163980	GSE76895	GSE9006	GSE78721
Age					
0-10				8	
10-20				26	
20-30			2	2	
30-40	3		2		
40-50	8	1	6		
50-60	7	8	18		
60-70		1	14		
70-80			22		
80-90			4		
Gender					
Male	7	6	39	16	56
Female	11	4	29	20	74

## Data Availability

The analyzed datasets generated during the study are available from the corresponding author on reasonable request.
